# Ruptured Posterior Inferior Cerebellar Artery Aneurysm Presenting With Cerebellar Intraparenchymal and Intraventricular Hemorrhage: A Case Report

**DOI:** 10.7759/cureus.101781

**Published:** 2026-01-18

**Authors:** Emmanuel Christy F Obien, Patricia Jarmin L Pua, Adrienne D Maglinao, Johnny K Lokin, Pedro Danilo J Lagamayo

**Affiliations:** 1 Department of Neuroscience and Behavioral Medicine, Section of Adult Neurology, University of Santo Tomas Hospital, Manila, PHL; 2 Department of Radiological Sciences, University of Santo Tomas Hospital, Manila, PHL; 3 Department of Surgery, Section of Neurosurgery, University of Santo Tomas Hospital, Manila, PHL; 4 College of Rehabilitation Sciences, University of Santo Tomas, Manila, PHL

**Keywords:** aneurysmal rupture, cerebellar hemorrhage, endovascular coiling, posterior circulation, posterior inferior cerebellar artery aneurysm

## Abstract

Aneurysmal subarachnoid hemorrhage (aSAH) may present with intraparenchymal and intraventricular extension, particularly in posterior circulation aneurysms, which warrants careful evaluation for secondary causes, including an underlying macrovascular lesion. We report the case of a 39-year-old woman who presented with a sudden-onset severe headache, nuchal pain, and transient loss of consciousness. Initial non-contrast cranial computed tomography demonstrated a dominant left cerebellar intraparenchymal hemorrhage with subarachnoid and intraventricular extension, accompanied by acute hydrocephalus. Given the patient’s age, clinical presentation, and hemorrhage pattern, computed tomography angiography was performed and revealed a ruptured wide-neck saccular aneurysm of the left posterior inferior cerebellar artery (PICA) with multiple daughter sacs.

The patient underwent successful endovascular coil embolization, preserving parent vessel patency, and made a complete neurological recovery. This case highlights a recognized but high-risk presentation of aneurysmal subarachnoid hemorrhage with cerebellar parenchymal extension and reinforces the importance of early vascular imaging in younger patients and in posterior fossa hemorrhage patterns. Prompt diagnosis and timely aneurysm securing remain critical determinants of the outcome.

## Introduction

Ruptured posterior inferior cerebellar artery (PICA) aneurysms account for only 0.5% to 3% of all intracranial aneurysms [1,2]. Younger patients presenting with intraparenchymal hemorrhage involving the posterior fossa warrant prompt evaluation for secondary causes, including underlying macrovascular lesions. We present a case of a 39-year-old Filipino female with sudden-onset severe nape pain, headache, and loss of consciousness. Initial non-contrast cranial CT scan showed extensive intraparenchymal and extra-axial hemorrhages. The patient was initially managed as a case of left cerebellar intraparenchymal hemorrhage. However, further evaluation with CT angiography revealed a ruptured large left PICA saccular aneurysm. Medical stabilization and endovascular coiling were performed, resulting in a successful outcome. In this case report, we emphasize an unusually extensive hemorrhagic presentation of a ruptured PICA aneurysm and the importance of medical and surgical management to achieve the best outcomes.

## Case presentation

The patient was a 39-year-old female with no known comorbidities who presented initially with a sudden onset of severe nuchal pain, followed by bilateral occipital and temporal headache, associated with visual dimming and non-projectile vomiting. She subsequently experienced a loss of consciousness, which prompted an Emergency Department (ED) consultation. Upon regaining consciousness, the patient exhibited post-syncopal confusion, diplopia, and a persistent headache. Past medical history was unremarkable. However, there was a family history of stroke and hypertension. Personally and socially, there was a significant smoking and drinking history, which she stopped 12 years ago. At the ED, blood pressure was elevated at 150/90 mmHg. Further neurologic examination revealed left lateral rectus palsy, bidirectional nystagmus, and left upper extremity dysmetria, with no meningeal signs of irritation.

**Figure 1:**
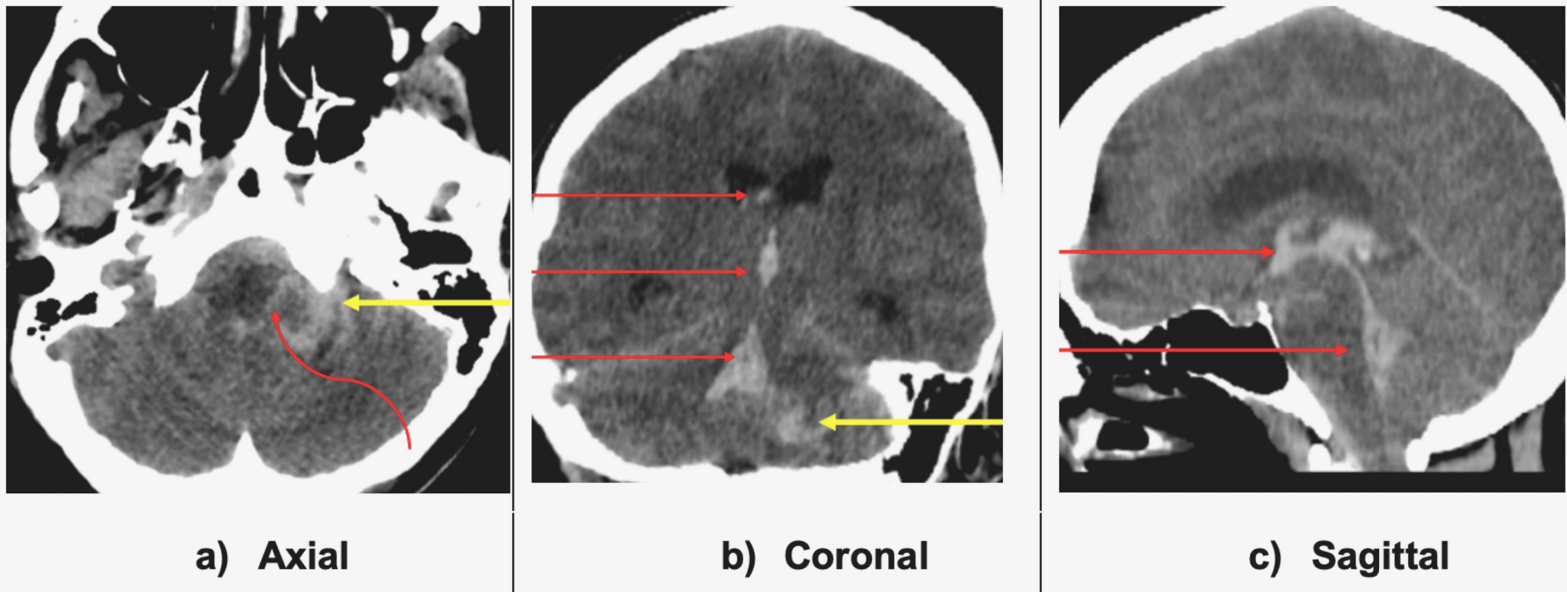
Non-contrast cranial CT scan Acute intraparenchymal left cerebellar hemorrhage (yellow arrows in A and B) with extension to the ventricles (straight red arrows in B and C) via the foramen of Luschka (curved red arrow in A) and subarachnoid cisterns.

A non-contrast cranial CT scan (Figure 1) demonstrated an intraparenchymal hyperdense fluid collection in the anterior left cerebellum extending to the subarachnoid cisterns and the ventricles via the foramen of Luschka. The collection showed intraventricular extension up to the lateral ventricles with resultant ventricular dilatation. Periventricular hypodensities, consistent with trans-ependymal seepage of fluid, were noted and reflective of acute hydrocephalus.

Medical management with nimodipine, levetiracetam, mannitol, and paracetamol was started.

**Figure 2:**
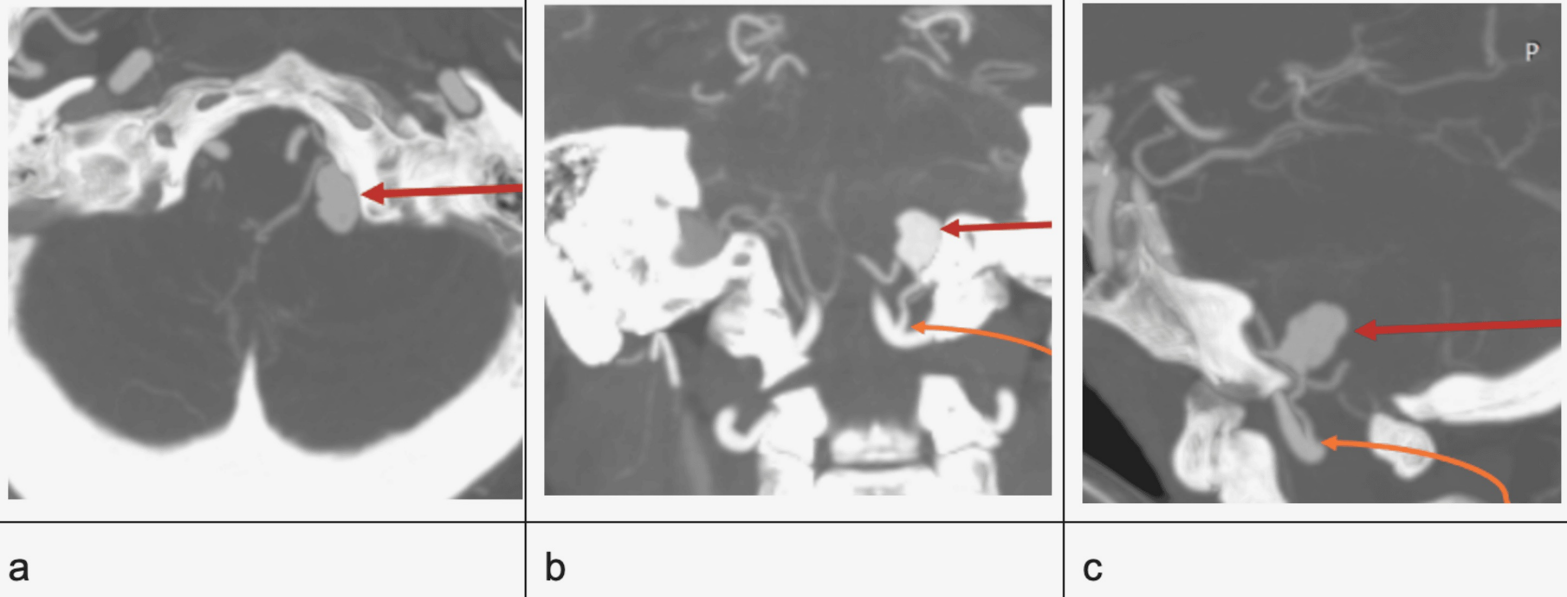
CT angiography in maximum intensity projection (MIP) reconstruction Wide neck saccular aneurysm (red arrows) in the left distal posterior inferior cerebellar artery arising from the left vertebral artery (orange arrows in b and c) seen in axial (A), coronal (B), and en-profile view on sagittal image.

Preliminary CT findings prompted further evaluation with CT angiography (Figure 2), which revealed a saccular aneurysm in the proximal left PICA, measuring about 0.9 x 1.0 cm (W x H) and with a neck of 0.6 cm. The aneurysm showed multiple teat formations and signs of rupture. Hyperdense fluid collections, reflective of acute hemorrhage, are seen in the subarachnoid spaces and left cerebellar parenchyma. Intraventricular extension with acute mild obstructive hydrocephalus is still evident. 

Neurosurgical referral for endovascular coiling was contemplated with the intent of permanent aneurysm obliteration, maintenance of parent artery patency, and preservation of neurological function.

**Figure 3:**
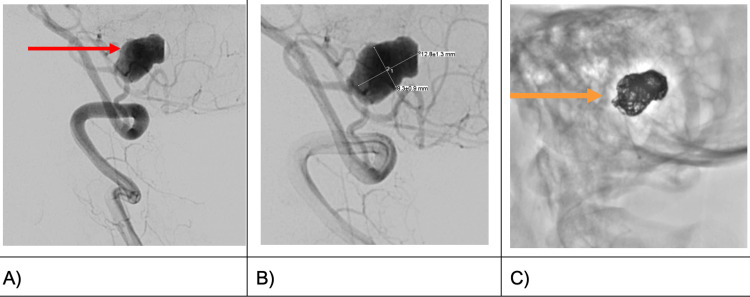
Digital subtraction angiography (DSA) images demonstrating a saccular aneurysm arising from the left PICA before (red arrow in A) and following (orange arrow in C) endovascular coiling PICA: posterior inferior cerebellar artery

Digital subtraction angiography (DSA) (Figure 3) confirmed the precise location of the aneurysm at the left posterior inferior cerebellar artery (PICA), measuring 1.3 cm x 0.8 cm (L x W) (Figure 3b), including the focal aneurysmal outpouching identified as the teat and exact point of rupture.

Endovascular embolization via a right common femoral artery access was performed by inserting microcatheters into the left vertebral artery, and ultimately into the left PICA. A total of six platinum-tungsten alloy detachable coils were utilized to achieve 95% (satisfactory) occlusion of the aneurysmal sac and preservation of distal flow.

The post-procedural course was unremarkable, with no new neurologic deficits. A three-day transcranial Doppler (TCD) ultrasound was done for vasospasm monitoring following complaints of persistent mild to moderate headache. The patient was managed medically, and subsequent TCD results were negative for vasospasm. Maintenance on a two-week course of anticoagulation with oral clopidogrel was done post-endovascular aneurysm coiling. The patient was eventually discharged on day 7 post-coiling. Follow-up after a month showed no recurrence of symptoms or new-onset focal deficits.

## Discussion

Posterior inferior cerebellar artery aneurysms are uncommon lesions and may present with a broad range of clinical and radiologic features, including subarachnoid hemorrhage with intraparenchymal and intraventricular extension. In such cases, early recognition of an underlying aneurysmal etiology is essential, as timely medical stabilization and definitive aneurysm treatment are critical determinants of outcome. In the following sections, we discuss the medical and surgical considerations that guided management in this patient, in accordance with current guideline-based recommendations.

Management

The patient was managed medically and surgically in accordance with the latest American Heart Association (AHA)/American Stroke Association (ASA) subarachnoid hemorrhage (SAH) guidelines (2023) [3]. Medical stabilization was initiated first by admitting our patient to the intensive care unit. Headache control required the use of round-the-clock pain paracetamol and non-steroidal anti-inflammatory drugs (NSAIDs; ketorolac and celecoxib), nausea and vomiting with metoclopramide, management of increased intracranial pressure with mannitol, prevention of delayed cerebral ischemia (DCI) with enteral nimodipine, control of blood pressure (BP) to systolic value of less than 140 mmHg with the use of nicardipine drip, levetiracetam for prophylactic seizure control due to intraparenchymal extension of the bleeding, and routine serial neurologic status monitoring. BP control is vital in the management of patients with a ruptured aneurysm to prevent re-rupture. The 2023 AHA/ASA guidelines emphasize individualized blood pressure management prior to aneurysm securing, avoiding severe hypertension while maintaining adequate cerebral perfusion [3]. However, meta-analyses of factors predictive of early rebleeding in aneurysmal SAH suggested higher rates in patients with systolic BP>160 mmHg, but not in those with systolic BP <140 mmHg. Hence, our team opted to target the latter systolic value. While the patient underwent medical stabilization, endovascular surgery was contemplated, and the choice of intervention was coiling of the aneurysm.

Surgical approach

The rarity of ruptured PICA aneurysms in the posterior circulation persists as a management predicament despite the various advancements in both microsurgical and endovascular techniques.

Surgical intervention for proximal PICA aneurysms, which are located near the brainstem and cerebellum, carries an inherent risk of lower cranial nerve injury [4]. Endovascular coiling is the procedure of choice for ruptured PICA aneurysms. Endovascular coiling aims to obliterate aneurysms while maintaining parent artery patency and preserving neurological function, as was the intent in the case presented. Open surgery would require careful navigation and exposure near the brainstem, which may pose a risk of further neurological collateral damage. Endovascular techniques can reach these aneurysms intraluminally, which may lessen the need for extensive surgical dissection and retraction of sensitive brain tissue.

Outcomes depend on three factors according to various literature: anatomic variations, aneurysm morphology, and patient-specific factors [4].

Anatomic variations

The PICA exhibits an intricate and variable anatomical course among the cerebellar arteries. This vessel is characterized by two loops and five distinct segments, which vary anatomically [5]. These segments are defined based on their specific relationships with the brainstem and cerebellum. A PICA aneurysm can arise either proximal or distal to the vertebral artery. The majority of aneurysms arise from the junction of the vertebral artery take-off. The clinical impact of a ruptured aneurysm in this location leads to extensive subarachnoid hemorrhage (SAH) in the basal cisterns and fourth ventricle, leading to acute hydrocephalus and brainstem compression, clinically presenting with loss of consciousness due to ascending reticular activating system involvement. Parenchymal involvement leads to cerebellar and brainstem compression, resulting in ataxia, dysmetria, and cranial nerve palsies, sometimes tonsillar herniation. In our case, the subarachnoid hemorrhage expanded through the foramen of Luschka and into the ventricles. The high-pressure trajectory of blood from the ruptured aneurysm may have dissected the cerebellar parenchyma, leading to intracerebellar hemorrhage. In contrast, the direction of blood flow relative to the anatomy may have led to intraventricular extension.

Aneurysm morphology

The morphology of a PICA aneurysm influences a patient's outcome due to its impact on adjacent structures, treatment challenges, and potential neurological damage. In terms of size, large aneurysms (>12 mm) are associated with a higher risk of rupture and lead to much more severe hemorrhage, manifesting as intraparenchymal bleed and intraventricular extension, as in our case [6]. This leads to increased intracranial pressure and brainstem compression, resulting in poorer outcomes. Size alone, however, is not the only predictor of rupture, because smaller aneurysms may also cause rapid neurologic deficits depending on their location. In terms of type, aneurysms can be saccular, fusiform, dissecting, or mixed. Saccular aneurysms have a higher risk of re-rupture with a poorer prognosis. Our case had a large saccular aneurysm (12.5 mm) with multiple teat formations, posing a supposed poorer outcome.

Patient-specific factors

Four patient-specific factors that may predict outcomes have been identified: age, presentation, comorbidities, and complications [7]. 

Age

Older people beyond 40 years old have a higher predisposition for rupture of intracranial aneurysms, contrary to our patient. This is due to an interplay of factors, including cumulative hemodynamic stress, degenerative changes in the vessel walls, and the increased prevalence of comorbidities. The risk factors of the patient, particularly smoking, although more than a decade ago, could have contributed to the cumulative vascular stress that altered the vessel walls, which led to a predisposition for aneurysmal formation and rupture.

Presentation

The presenting deficits of the patient, particularly diplopia and left lateral rectus palsy, could be a strong localizing sign of a ruptured PICA aneurysm. The lateral rectus muscle is subserved by the abducens nerve, which has a long intracranial course after exiting the brainstem at the pontomedullary junction. At this point, the nerve is vulnerable to stretching and compression brought by an increased ICP and subsequent displacement of the brainstem. While sometimes considered a "false localizing sign" of generalized raised ICP, the brainstem effects can be more direct in the context of a posterior fossa lesion.

Comorbidities

Comorbidities such as hypertension and diabetes are the most critical prognostic factors, as they place a higher risk of spontaneous intracranial aneurysm rupture. However, in our case, there were no comorbidities at the time of symptom onset. Rupture of the aneurysm in this case is likely due to the inherent structural weakness of the aneurysm wall and other underlying risk factors that predispose to the condition, particularly prior smoking history. Despite having no identified co-morbidities, risk factors play a role in the aneurysmal rupture in our patient.

Complications

Acute hydrocephalus after aneurysmal SAH, especially in the posterior fossa, connotes poorer patient outcomes. Our patient has a modified Fisher Grade Score of IV. While not strictly a patient factor, higher Fisher grading is a strong predictor of poor outcomes. The occurrence of intraventricular hemorrhage and intracerebellar hemorrhage is a significant negative prognostic indicator. 

Despite initial successful treatment of the aneurysm, DCI secondary to vasospasm is a significant cause of poor outcomes and neurological deficits [7]. While PICA aneurysms have a lower incidence of severe angiographic vasospasm compared to aneurysms in the anterior circulation, local posterior circulation vasospasm can still be significant. A surveillance transcranial Doppler ultrasound monitoring was done postoperatively in our patient to check for vasospasm [7]. Results were unremarkable, and nimodipine, a calcium-channel blocker, was administered for 21 days. Seizures may also occur as a complication of SAH, and they may impact overall recovery. In our case, prophylactic seizure control with levetiracetam was administered due to intraparenchymal extension, hydrocephalus, and high-grade modified Fisher score. The guidelines recommend a short course of treatment for patients who are labeled as high risk for seizure events [7].

## Conclusions

Ruptured posterior inferior cerebellar artery aneurysms may present with cerebellar intraparenchymal and intraventricular hemorrhage in addition to classic subarachnoid bleeding, a pattern that is well-recognized in aneurysmal subarachnoid hemorrhage and should prompt early vascular imaging. This case report highlights that in patients with high-risk clinical features, including young age at onset, posterior fossa hemorrhage, and absence of longstanding hypertension, evaluation for an underlying macrovascular lesion with early computed tomography angiography and confirmatory digital subtraction angiography is essential to identify surgically treatable causes and guide timely intervention. This case underscores that prompt recognition, appropriate medical stabilization, and early aneurysm securing can result in favorable outcomes despite the high hemorrhage burden and unfavorable aneurysm morphology.
